# Supplementary material for: Dysconnection and cognition in schizophrenia: A spectral dynamic causal modeling study
Source: Hum Brain Mapp. 2023 Feb 28;44(7):2873–96. doi: 10.1002/hbm.26251 (PMC10089110; doi:10.1002/hbm.26251)
Supplement: Supplementary file 1 — Appendix S1: Supplementary information [file HBM-44-2873-s001.docx]

Supplementary Material

**Dysconnection and cognition in schizophrenia: a spectral DCM study**

Tahereh S. Zarghami^1,2^, Peter Zeidman^3^, Adeel Razi^3,4,5,6^,

Fariba Bahrami^1,2^, Gholam-Ali Hossein-Zadeh^1^

1. Bio-Electric Department, School of Electrical and Computer Engineering, College of Engineering, University of Teran, Tehran, Iran

2. Human Motor Control and Computational Neuroscience Laboratory, School of Electrical and Computer Engineering, College of Engineering, University of Tehran, Tehran, Iran

3. The Wellcome Centre for Human Neuroimaging, University College London, Queen Square, London WC1N 3AR, UK

4. Turner Institute for Brain and Mental Health, Monash University, Clayton, VIC

5. Monash Biomedical Imaging, Monash University, Clayton, VIC

6. CIFAR Azrieli Global Scholars Program, CIFAR, Toronto, Canada

Table S1: Frequency of inclusion of the top EC and MCCB variables returned by bootstrap aggregation, as part of the ensemble feature selection procedure. Arrows (=>) indicate direction of connection. The last column shows whether the EC parameter had shown diagnostic effect in the group analysis. Abbreviations: SC, subcortical; AUD, auditory; SM, sensorimotor; VIS, visual; COG, cognitive control; DMN, default mode network; CB, cerebellum.

| **EC parameters** | **Frequency of inclusion** | **Network** | **Diagnostic** |
| --- | --- | --- | --- |
| SMA => SMA | 0.76 | SM | 0 |
| ParaCL1 => ParaCL1 | 0.185 | SM | 1 |
| PHG => ITG | 0.18 | COG | 0 |
| R.STG+IFG => R.STG+IFG | 0.175 | COG | 0 |
| PHG => aInsula | 0.17 | COG | 0 |
| L-PoCG => ParaCL1 | 0.155 | SM | 0 |
| SMA => MCC | 0.155 | COG | 0 |
| R.STG+IFG => aInsula | 0.155 | COG | 0 |
| ITG => SMA | 0.155 | COG | 0 |
| MiFG1 => MiFG1 | 0.145 | COG | 0 |
| MiFG1 => PHG | 0.14 | COG | 1 |
| L-AG => ACC | 0.13 | DMN | 0 |
| L-MOG => Cuneus1 | 0.125 | VIS | 0 |
| CB => L-CB | 0.125 | CB | 0 |
| Caudate => Thalamus | 0.12 | SC | 0 |
| ParaCL2 => ParaCL1 | 0.12 | SM | 0 |
| MTG => LingualG | 0.12 | VIS | 0 |
| SMA => IFG | 0.12 | COG | 0 |
| PoCG => PoCG | 0.115 | SM | 1 |
| R-IPL => MiFG1 | 0.115 | COG | 0 |
| pInsula => MCC | 0.115 | COG | 1 |
| PCC1 => PCC1 | 0.115 | DMN | 0 |
| STG2 => STG2 | 0.11 | AUD | 1 |
| **MCCB score** | **Frequency of**  **inclusion** |  |  |
| Social cognition | 1 |  |  |
| Reasoning/problem solving | 0.975 |  |  |
| Working memory | 0.935 |  |  |
| Attention/vigilance | 0.565 |  |  |
| Verbal learning | 0.555 |  |  |
| Speed of processing | 0.53 |  |  |
| Visual learning | 0.27 |  |  |


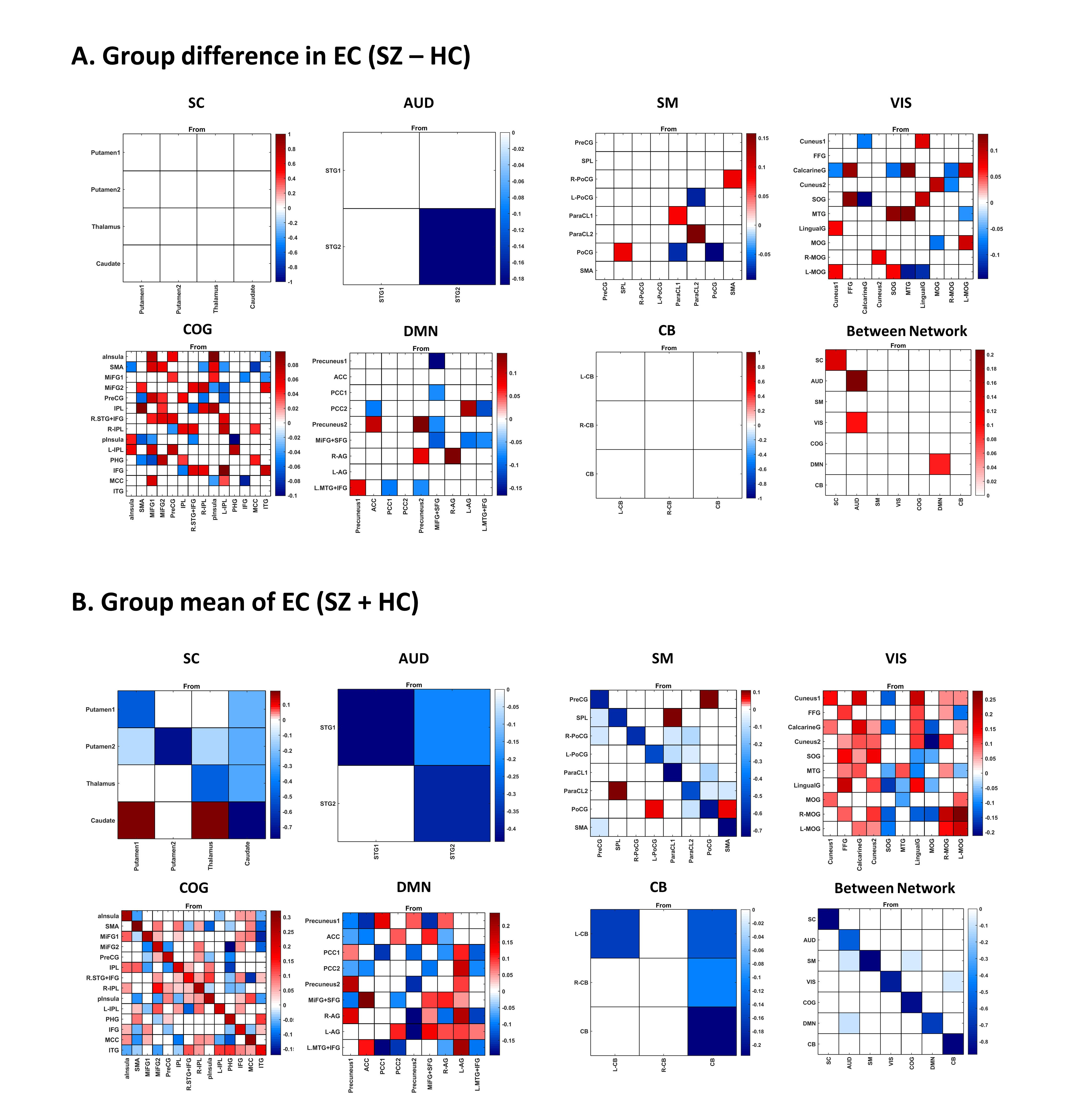


Fig. S1: Expected posteriors of group EC effects, adjusted for age, gender and medication dosage. (A) Group differences in effective connectivity (SZ – HC). (B) Group mean of effective connectivity across all subjects. Each entry 𝐴𝑖𝑗 denotes the expected group effect from (sub)network 𝑗 to 𝑖. Only significant entries (95% credible interval not containing zero) have been colored. Diagonal entries encode inhibitory self-connections as log scaling parameters that can be converted to units of Hz using $-0.5*exp(A_{ii})$. Hence, more positive diagonal entries denote higher self-inhibition in the patient group. Network and subnetwork abbreviations are available in Table 2 of the main text.


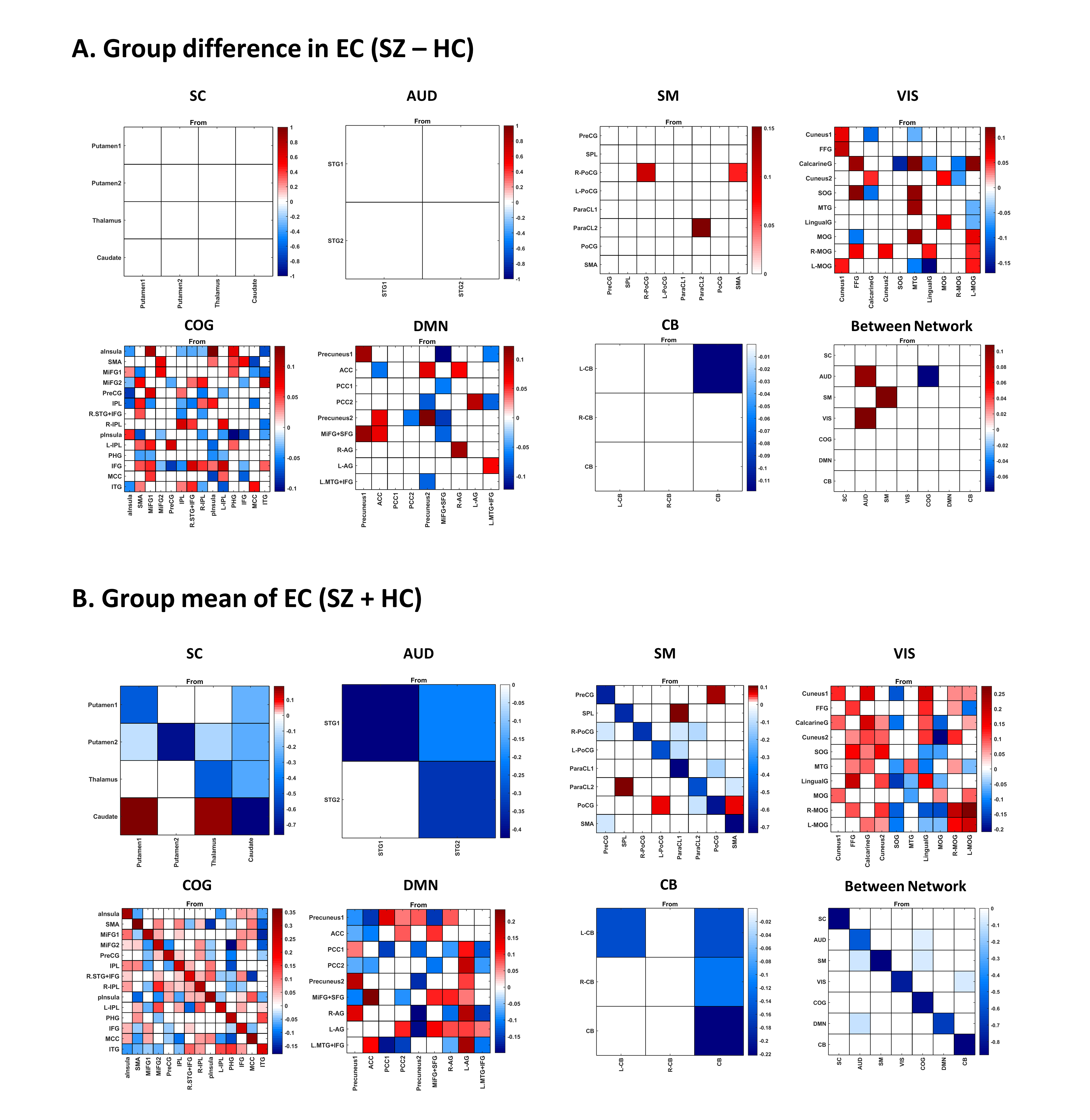


Fig. S2: Expected posteriors of group EC effects, adjusted for age and gender, but not medication dosage. (A) Group differences in effective connectivity (SZ – HC). (B) Group mean of effective connectivity across all subjects. Each entry 𝐴𝑖𝑗 denotes the expected group effect from (sub)network 𝑗 to 𝑖. Only significant entries (95% credible interval not containing zero) have been colored. Diagonal entries encode inhibitory self-connections as log scaling parameters that can be converted to units of Hz using $-0.5*exp(A_{ii})$. Hence, more positive diagonal entries denote higher self-inhibition in the patient group. Network and subnetwork abbreviations are available in Table 2 of the main text.


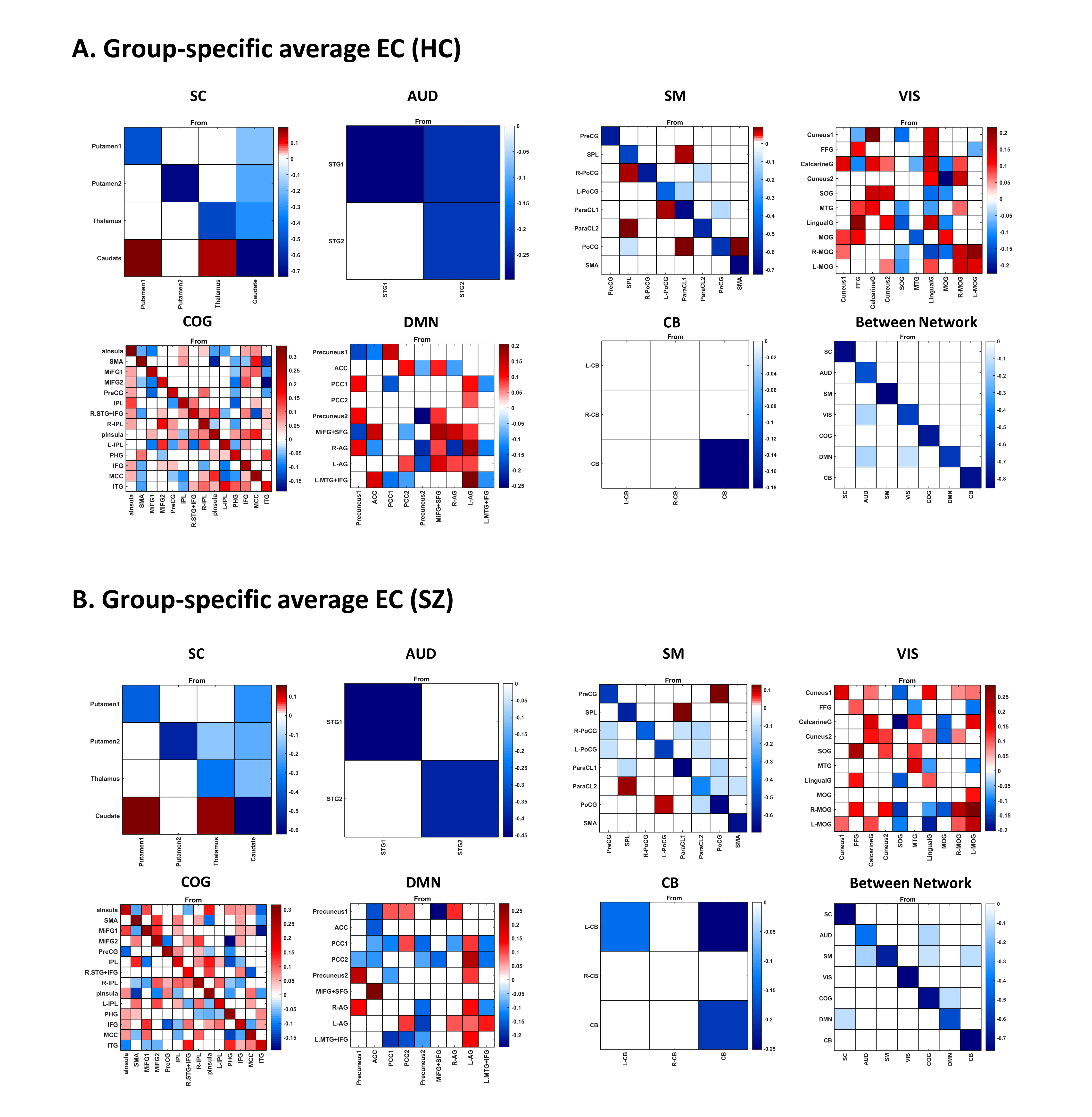


Fig. S3: Expected posteriors of group-specific EC effects, adjusted for age and gender. (A) Average EC for the HC group. (B) Average EC for the SZ group. Each entry $A_{ij}$ denotes the expected group effect from (sub)network $j$ to $i$. Only significant entries (95% credible interval not containing zero) have been colored. Diagonal entries encode inhibitory self-connections as log scaling parameters that can be converted to units of Hz using $-0.5*exp(A_{ii})$. Network and subnetwork abbeviations are available in Table 2 of the main text.


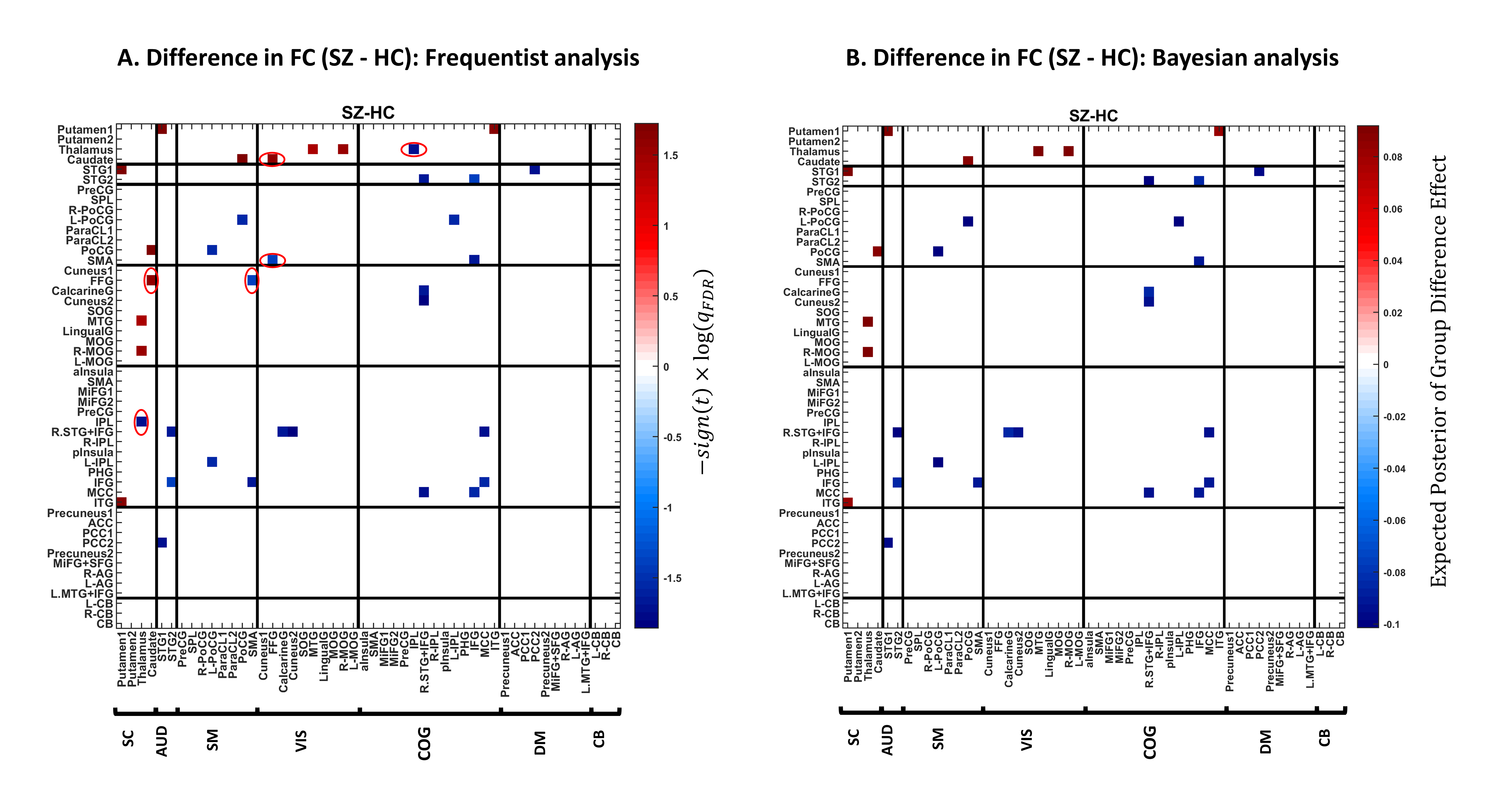


Fig. S4: Frequentist vs. Bayesian FC results (A) Significant group differences in FC (SZ-HC) estimated using the frequentist linear regression model outlined in section 2.4 (FC ~ 1 + diagnosis + age + gender + medication dosage). The p-values were adjusted using FDR correction. This is the same plot shown in Fig. 3-C of the main text. (B) Significant FC differences estimated using a Bayesian linear regression model (with the same design matrix, $X$, as the frequentist model) and precise null priors: $FC=X*\beta+e$ ;$e \sim N\left( 0,\sigma_{e}^{2} \right)$; $\sigma_{e}^{2}\sim InvGamma \left( a=3,b=0.5 \right)$; $\beta|\sigma_{e}^{2}\sim N\left( \mu=0,\sigma_{\beta}^{2}=0.1*\sigma_{e}^{2} \right)$. FC values were Fisher z-transformed (*atanh*) to comply with the normality assumptions. The model was set up and estimated using *bayeslm* and *estimate* functions in MATLAB 2021b. Significance was assessed based on 95% credible intervals of the posterior distributions. The results show that the Bayesian analysis has adjusted for multiple comparisons more stringently than the frequentist FDR correction (hence, the connections circled in red are not significant with Bayesian standards). This more conservative nature of Bayesian (relative to frequentist) credible intervals has been theoretically demonstrated in (Gelman et al., 2012; Gelman and Tuerlincks, 2000).


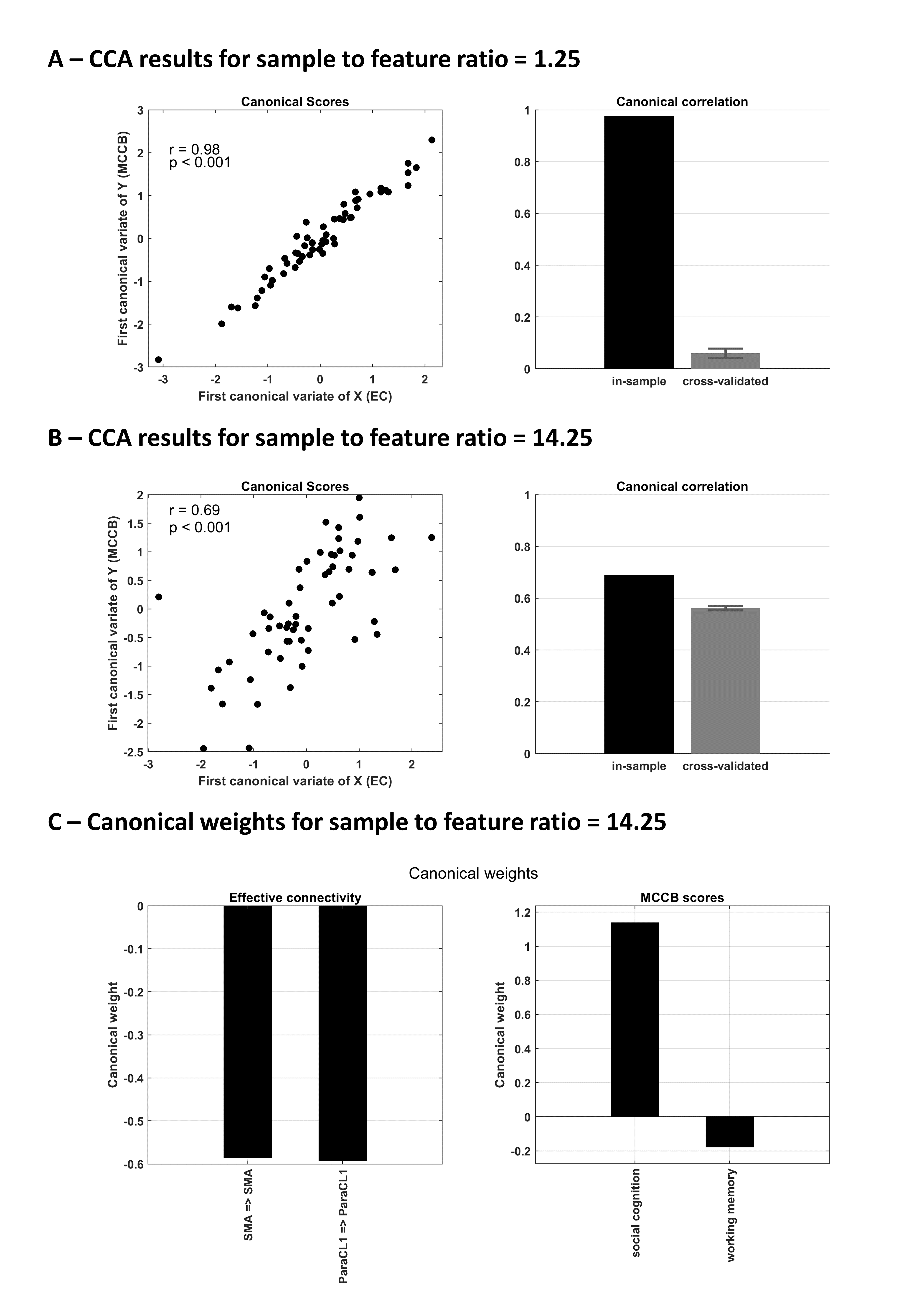


Fig. S5: Canonical correlation analysis between EC parameters and cognitive (MCCB) scores of SZ patients, for different sample to feature ratios (STFR). (A) Left: The first pair of canonical variates/scores plotted against each other. Right: in-sample and cross-validated estimates of the first canonical correlation, for STFR = 57/47 = 1.25. (B) CCA results for STFR = 57/4 = 14.25. (C) The (standardized) canonical weights for the CCA in panel B. Notably, the top EC and MCCB features match those in the main text (Fig. 7; STFR = 9.7), even though the ensemble feature selection procedure was repeated afresh (see section 2.6.2 of the main text for details). These results show that as STFR increases, the model becomes more generalizable, and the in-sample and cross-validated canonical correlation estimates get closer (Helmer et al., 2020; Yang et al., 2021).


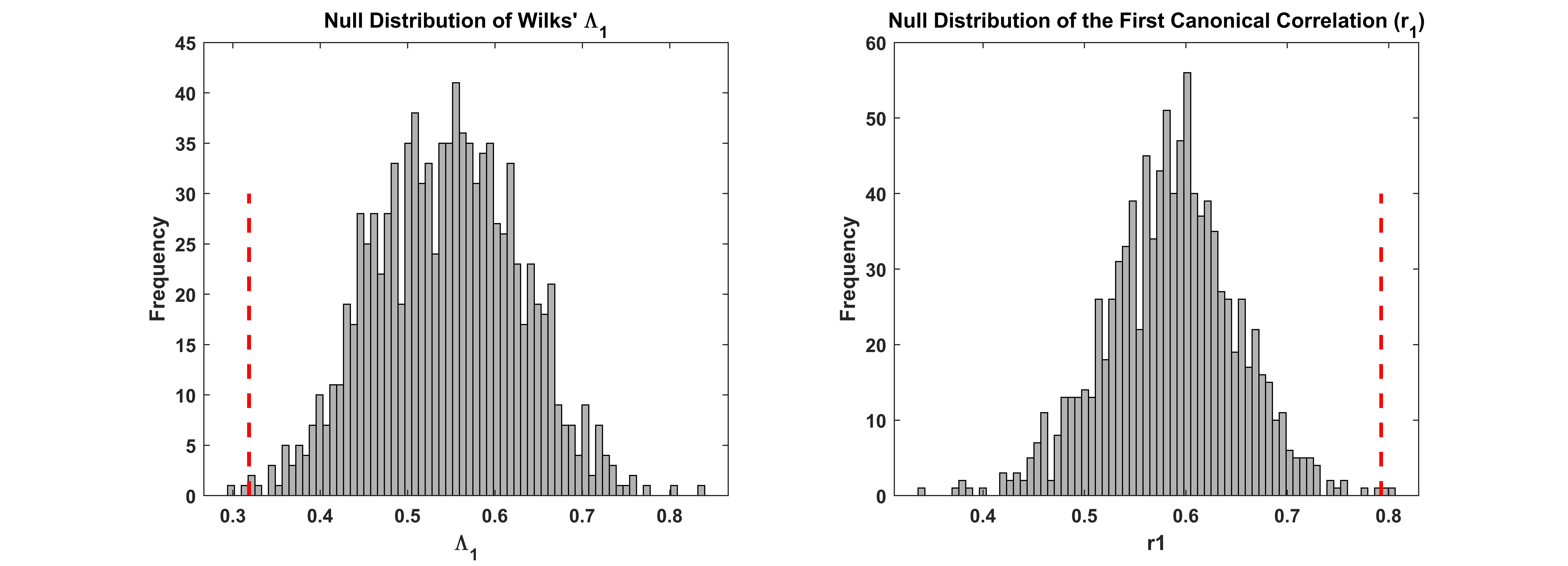


Fig. S6: Permutation-based inference for CCA between EC parameters (X) and MCCB scores (Y). (Left): Null distribution of Wilks’ lambda ($\Lambda_{1}$). (Right): Null distribution of the first canonical correlation ($r_{1}$). Null distributions were obtained by randomly permuting the rows of X (or Y) 1000 times, repeating the ensemble feature selection plus CCA, and recording the associated $\Lambda_{1}$ and $r_{1}$ (Dinga et al., 2021; Winkler et al., 2020). The vertical dashed lines mark the original values for unpermuted data ($\Lambda_{1}^{\mathrm{orig}}=0.32$ and $r_{1}^{orig}=0.79$). Permutation-based p-value is computed as the proportion of $\Lambda_{1}'s\leq$ $\Lambda_{1}^{\mathrm{orig}}$. In this case, p = 0.002, which is less optimistic than the parametric p-value (<0.001), as explained in (Dinga et al., 2021).
